# Age-Dependent Maturation of Toll-Like Receptor-Mediated Cytokine Responses in Gambian Infants

**DOI:** 10.1371/journal.pone.0018185

**Published:** 2011-04-13

**Authors:** Sarah Burl, John Townend, Jainaba Njie-Jobe, Momodou Cox, Uche J. Adetifa, Ebrima Touray, Victoria J. Philbin, Christy Mancuso, Beate Kampmann, Hilton Whittle, Assan Jaye, Katie L. Flanagan, Ofer Levy

**Affiliations:** 1 Infant Immunology, Medical Research Council (UK) The Gambia, Fajara, The Gambia; 2 Department of Paediatrics, Imperial College London, Paddington, London, United Kingdom; 3 Statistics Department, Medical Research Council (UK) The Gambia, Fajara, The Gambia; 4 Division of Infectious Diseases, Department of Medicine, Children's Hospital Boston and Harvard Medical School, Boston, Massachusetts, United States of America; University of Cambridge, United Kingdom

## Abstract

The global burden of neonatal and infant mortality due to infection is staggering, particularly in resource-poor settings. Early childhood vaccination is one of the major interventions that can reduce this burden, but there are specific limitations to inducing effective immunity in early life, including impaired neonatal leukocyte production of Th1-polarizing cytokines to many stimuli. Characterizing the ontogeny of Toll-like receptor (TLR)-mediated innate immune responses in infants may shed light on susceptibility to infection in this vulnerable age group, and provide insights into TLR agonists as candidate adjuvants for improved neonatal vaccines. As little is known about the leukocyte responses of infants in resource-poor settings, we characterized production of Th1-, Th2-, and anti-inflammatory- cytokines in response to agonists of TLRs 1-9 in whole blood from 120 Gambian infants ranging from newborns (cord blood) to 12 months of age. Most of the TLR agonists induced TNFα, IL-1β, IL-6, and IL-10 in cord blood. The greatest TNFα responses were observed for TLR4, -5, and -8 agonists, the highest being the thiazoloquinoline CLO75 (TLR7/8) that also uniquely induced cord blood IFNγ production. For most agonists, TLR-mediated TNFα and IFNγ responses increased from birth to 1 month of age. TLR8 agonists also induced the greatest production of the Th1-polarizing cytokines TNFα and IFNγ throughout the first year of life, although the relative responses to the single TLR8 agonist and the combined TLR7/8 agonist changed with age. In contrast, IL-1β, IL-6, and IL-10 responses to most agonists were robust at birth and remained stable through 12 months of age. These observations provide fresh insights into the ontogeny of innate immunity in African children, and may inform development of age-specific adjuvanted vaccine formulations important for global health.

## Introduction

The greatest burden of morbidity and mortality from infectious diseases occurs in children under 5 years of age, with the highest rates occurring in resource-poor countries. This urgent global issue is the target of United Nations Millennium Goal 4 to reduce under-five child mortality by two-thirds by 2015 [1]. One of the most effective measures to prevent infection is vaccination early in life, in particular at birth as it is the most reliable point of healthcare contact [2], [3]. However, young children often do not respond to vaccines as efficiently as adults as a result of distinct features of their immune systems [4]–[8].

Among the distinct features of neonatal immune system is a diminished ability of monocytes and antigen-presenting cells (APCs) to generate Th1-polarizing signals in response to most stimuli, including reduced production of TNFα and interferon gamma (IFNγ) important for host defence against intracellular pathogens and for generation of adaptive immune responses [9]–[11]. However, the impairment is stimulus-specific and some stimuli are able to effectively activate neonatal APCs [7], [10], [12]. In this context, characterizing the ontogeny of human immune responses including the contribution of innate immunity, provides insight into susceptibility of newborns and infants to infection, assesses immune polarization that may affect risks of allergy and atopy [13], and may inform development of age-specific adjuvanted vaccines [7].

Modifying the innate response may increase the strength of adaptive responses required for inducing efficient immunological memory in infants. Until recently, the only approved adjuvant component in vaccines was alum. However, the discovery that microbial products activate host cells via pattern recognition receptors such as Toll-like receptors (TLRs) and enhance adaptive immune responses by triggering cytokine production and dendritic cell maturation, has led to TLR agonists being developed as vaccine adjuvants [14]–[18]. The TLR4 agonist monophosphoryl lipid A (MPL) has been combined with alum (AS04) in hepatitis B and human papilloma vaccines[19]–[21]. Agonists of several other TLRs, including TLR3, 7, 8 and 9, are in clinical development as vaccine adjuvants targeting mycobacterial, parasitic and diseases [22] [7], [23]–[27]. Of note, several existing vaccines trigger TLRs: *Bacillus Calmette Guerin* (BCG), the most widely used vaccine against TB with an established safety and efficacy profile activates TLR2, -4 and -8 [28]–[30] and the meningococcal outer membrane protein complex used to adjuvant the Haemophilus conjugate vaccine, was subsequently shown to be a TLR2 agonist [31]. These examples suggest that in certain contexts TLR agonists can be safe and effective as vaccine adjuvants and highlight the importance of characterizing ontogeny of TLR-mediated responses in target populations, including newborns and infants.

Age-dependent changes in TLR function have been demonstrated in humans by natural deficiencies in TLR signaling molecules including a deficiency in IRAK-4 (a kinase involved in the TLR signaling cascade) [32], [33] and MyD88 (a TLR adaptor molecule) [11] that can result in life-threatening infections in infancy and childhood. Of note, infections decrease in IRAK-4-deficient children >8 years of age, suggesting a greater dependency early in life on TLR signaling for protection against infection. Although newborns demonstrate similar basal expression of monocyte TLRs at birth [34], neonatal blood mononuclear cell responses to TLR agonists are distinct from those of adults, with impairment of Th1 cytokine responses (e.g., TNFα, IFNγ) but similar or greater production of cytokines with Th2 (IL-6, IL-1β), Th17 (IL-23) and anti-inflammatory (IL-10) activity [4], [34]–[42]. By contrast, cord blood responses to TLR8 agonists, including imidazoquinoline compounds and single stranded RNAs (ssRNAs), induced robust adult-like TNFα responses raising the possibility that TLR8 agonists may serve as effective vaccine adjuvants for newborns and young infants [34], [43].

Because infant peripheral blood is more difficult to obtain than cord blood, relatively less is known about TLR function during infancy. The few infant studies available have been conducted in resource-rich settings and indicate that there is a marked increase in TLR-mediated Th1 cytokine production within the first 6 months of life [13], [37], [44], [45]. To our knowledge, there are no published studies of TLR function in newborns and infants from resource-poor countries and none from the African continent. Such data may be of considerable importance as these populations are at high risk of infection, and may demonstrate distinct innate response profiles compared to other populations with lesser burdens of disease. For example, infants from resource-poor countries often respond quite differently to immune stimuli including to BCG vaccination [46], [47]. In addition to providing insight into age-dependent development of TLR-mediated innate immunity, characterizing the ontogeny of TLR responses in an African country may also inform development of adjuvanted vaccines for high-risk newborns and infants. We therefore characterised the production of Th1, Th2, and anti-inflammatory cytokines in response to agonists of TLRs 1 – 9 in whole blood cultures derived from Gambian infants during the first year of life. We found that cord blood Th1-polarising cytokine responses were generally impaired, that TLR8 agonists gave the strongest Th1-cytokine responses at birth and that by 1 month of age the pro-inflammatory responses to most TLR agonists were increased, with predominance of responses to TLR4, -5 and -8 agonists that changed across the first 12 months of life.

## Materials and Methods

### Study Design

This cross-sectional study was approved by the Joint Gambia Government/Medical Research Council Ethics Committee and the London School of Hygiene and Tropical Medicine Ethics Committee. Between June and November 2009, 120 Gambian infants, up to 12 months of age, were recruited at the Sukuta Health Centre (a peri-urban (area surrounding an urban town with characteristics of a rural setting) population 30 minutes from the coast of The Gambia). Infants were recruited into 8 age groups: birth and 1-, 2-, 3-, 4-, 6-, 9- or 12- months of age (or +2 weeks of age stated; n = 15 per group). For every study subject, written informed consent of a parent/guardian was obtained. Children were excluded if they had any signs of intercurrent infection. Neonates were also excluded if they had a low birth weight (< 2.5 kg) or were a twin. Each child within an age group had received comparable vaccines according to the Gambian Extended Programme of Immunisation (EPI) schedule, and had not received any vaccine within the previous 7 days. Infants were also excluded if their weight was outside the target range for age stated on the Infant Welfare Card. Ten millilitres of umbilical cord blood (collected before delivery of the placenta) and 3 mL of infant venous blood was collected into tubes containing heparin (sodium salt from porcine intestinal mucosa, Sigma-Aldrich, Poole, UK) at 7.5 U/mL blood and transferred to the laboratories within 6 hrs of collection. Blood was acquired prior to administration of any EPI vaccine that may have been indicated on the day of recruitment.

### Cell culture conditions

From each blood sample, 100 µl of undiluted whole blood (per condition) was cultured overnight (18–24 hours) without stimulation (negative control), with phorbol 12-myristate 13-acetate (PMA; 0.1 µg/mL, Sigma)/ionomycin (calcium salt, from *Streptomyces conglobatus*, 1 µg/mL, Sigma) (positive control) and with each of the 9 TLR agonists (Table S1). TLR agonists were purchased from InvivoGen (San Diego, CA, US) and included: Pam3CSK4 (synthetic tripalmitoylated lipopeptide that mimics bacterial lipoproteins; TLR1/2, 1 µg/mL), Poly (I:C) (a synthetic analog of double-stranded RNA (dsRNA) associated with viral infection; TLR3, 100 µg/mL), LPS (*Escherichia. coli K12* Lipopolysaccharide; TLR4, 1 µg/mL), flagellin (from *Salmonella typhimurium*; TLR5, 10 µg/mL), FSL-1 (synthetic lipoprotein of *Mycoplasma salivarium*; TLR2/6, 10 µg/mL), ssRNA40 (20-mer phosphothioate protected single-stranded RNA oligonucleotide; TLR8, 10 µg/mL), CL075 (thiazoloquinolone derivative; TLR8, 10 µg/mL), Gardiquimod™ (an imidazoquinoline amine analogue to guanosine; TLR7, 10 µg/mL), ODN M362 (synthetic type C unmethylated-CpG dinucleotide-containing oligonucleotide; TLR9, 1 µM) (Table S1). TLR concentrations were selected based on those that induced maximal cytokine responses from previous studies [34], [43] and from preliminary studies in 9 months old Gambian infants (unpublished data). After overnight culture, 100 µL of ice-cold serum-free RPMI media was added to the wells and centrifuged at 1,500 rpm for 10 mins. 120 µL of supernatant was collected and stored at −20°C for subsequent analysis. For RNA studies, 300 µL of whole blood were cultured in each of four conditions (unstimulated, ssRNA, Gardiquimod™ or CLO75) for 4 hours then added to 860 µL PaxGene lysing reagent (PreAnalytiX, Becton Dickinson, France) prior to storage at −70°C ahead of RNA extraction.

### Cytokine measurement

Supernatants were thawed and centrifuged at 1500 rpm for 5 mins to pellet any precipitation in the sample. Concentrations of TNFα, IL-1β, IL-6, IFNγ and IL-10 (Th1 cytokine kit, Bio-Rad, Hercules, California, US) were measured for all samples. The bead array assays were conducted according to the manufacturer's instructions (Bioplex Reagent kit, Bio-Rad) using 50 µL of serial (1∶4) dilutions of the standard in RPMI medium containing 10% human serum and 50 µl of samples. Standard curve outliers were eliminated by identifying samples where the coefficient of variance (CV) was greater than 10% and observed/expected x 100 (obs/exp*100) was outside the range of 100±20. Cytokine concentrations below the level of detection (out of range (OOR) <) were assigned a value of half the lowest value recorded in that assay. Similarly, all samples with values above the level of detection (OOR>) were assigned a value of twice the largest value recorded in that assay.

### RNA extraction

Samples were thawed and left at room temperature (15 – 25°C) >4 hours for complete lysis prior to centrifugation for 10 mins at 5000 rpm. RNA was extracted according to manufacturer's instructions with modification for the small blood volumes (PaxGene Blood RNA kit, QIAGEN, Germany). Briefly, the pellet was resuspended in 500 µL of RNase-free water and centrifuged again at 5,000 rpm for 10 mins. The pellet was then resuspended in 360 µL of BR1 buffer (PaxGene Blood RNA kit). The samples were further processed according to the manufacturer's instructions and eluted in a final volume of 80 µL of Tris/EDTA (TE)-based elution buffer (BR5). RNA was then purified further using the RNeasy Mini Elute Clean Up kit (QIAGEN, Germany) and resuspended in 12 µL of BR5. The RNA yield was measured on the Nanodrop (Thermo Scientific, Wilmington, Delaware, US) and purity was based on absorption values at 280 nm (protein) and 230 nm (detection of contaminating organics/proteins) using the following criteria: 260 nm/280 nm >1.8 and 260 nm/230 nm >1.8.

### SA Bioscience Microarrays

0.1 µg total RNA was reverse transcribed to cDNA using RT^2^ First Strand Kit (C-03) (SA Biosciences, Frederick, MD, US) according to the manufacturer's instructions and diluted with RNase-free water into a final volume of 111 µL. A customized 6-gene real time PCR array (SABiosciences, Frederick, MD) was employed, containing primers specific for TNF, IL-6, IL-10, IL12A, IL12B and IFNG and using the RT-SYBR Green/ROX qPCR Master Mix according to the manufacturer's instructions (SABiosciences, Frederick, MD) using approximately 1 ng of original RNA per gene. mRNA expression was calculated as a copy number (Ct) value and normalized using multiple housekeeping genes (B2M, RPL13A, GAPDH, ΔC_t_). Fold change (ΔΔC_t_) in gene expression was used to display the data and calculated between the unstimulated control cells in comparison to TLR agonist-stimulated cells applying the following equation: if X >1, X, -1/X, if X =  ΔC_t_ therefore all values <0 represented down-regulated genes and all values >0 represented up-regulated genes. ΔC_t_ values were used for all statistical analysis.

### Statistical analysis

Net cytokine responses were calculated by subtracting the cytokine concentrations in the medium control wells from those in supernatants derived form samples stimulated with TLR agonists. For each TLR agonist/cytokine combination the resulting datasets of net responses (all ages combined) were dichotomised into ≥ median value, or < median value. Due to a number of responses above the level of detection, comparisons of responses at difference ages were made by comparing the proportions of values above and below the median using Fisher's exact test. Tests for a trend with age were carried out using logistic regression with above or below the median as the response variable and age as the independent variable. Comparisons between the response to medium and the gross responses to each of the TLR agonists in cord blood were made using Sign tests which are robust to the influence of out of range values, in accordance with the paired nature of the data. Analyses that compared two time points or two ages were done using a non-parametric Mann U Whitney test and correlations were calculated on log-transformed data using a Pearson correlation. Data were analysed using Stata version 11.0 (StataCorp, Texas, US) and GraphPad Prism version 5.01 (GraphPad Software Inc., US). Real time PCR analysis used software from SA Biosciences as stated above. Box and whisker plots demonstrate inter-quartile ranges indicated by boxes, median values by horizontal bars, and 10 and 90 percentiles indicated by whiskers. P-values <0.05 were taken to indicate statistical significance.

## Results

### Reactivity to TLR4, 5 and 8 agonists predominate at birth

The positive control PMA/ionomycin induced cytokine responses that were largely independent of age (TNFα p = 0.603, IL-6 p = 0.758, IL-1β p = 0.257, IFNγ p = 0.167). The lone exception was IL-10, whose production diminished with increasing age (p = 0.005; data not shown).

Compared to the unstimulated control, there was a significant induction of TNFα, IL-6, IL-1β and IL-10 production in cord blood to all the TLR agonists studied with the exception of FSL-1 (TLR2/6) that did not induce a significant TNFα response (Table 1).

**Table 1:** TLR agonists induce cytokine production in cord blood of Gambian newborns.

	Unstim. Control	Pam. (TLR1/2)	PolyI:C (TLR3)	LPS (TLR4)	Flag. (TLR5)	FSL-1 (TLR2/6)	Guard. (TLR7)	ssRNA (TLR8)	CL075 (TLR7/8)	ODN (TLR9)
**TNFα**	1.309	3.98*	6.34*	1,117.17***	133.52***	4.29	12.13*	5,003.68***	5,180.04***	10.74**
**IL-6**	245.9	7,161.75***	1,831.22**	22,529.47***	25,209.28***	8,811.02***	5,390.73***	14,412.48**	21,262.00***	3,802.29**
**IL-1β**	3.11	36.42**	15.31**	1,293.61***	1,522.90***	30.17**	59.92**	3,069.16***	2,557.96***	36.92**
**IL-10**	10.36	151.20***	35.05**	966.90***	1,145.50***	145.06***	156.36***	745.06***	1,647.61***	33.06**
**IFNγ**	3.335	3.34	2.83	6.37	6.38	3.24	3.34	23.36	59.17**	3.34

100 µl cord whole blood was cultured overnight with each of the TLR agonists and cytokine production (pg/mL) in supernatants was measured as described in the Materials and Methods. Concentrations are absolute values in pg/mL. Comparisons between unstimulated and stimulated values, n = 12**–**15; *p = 0.05 – 0.019, **p = 0.01 – 0.001, ***p<0.001.

**Figure 1:**
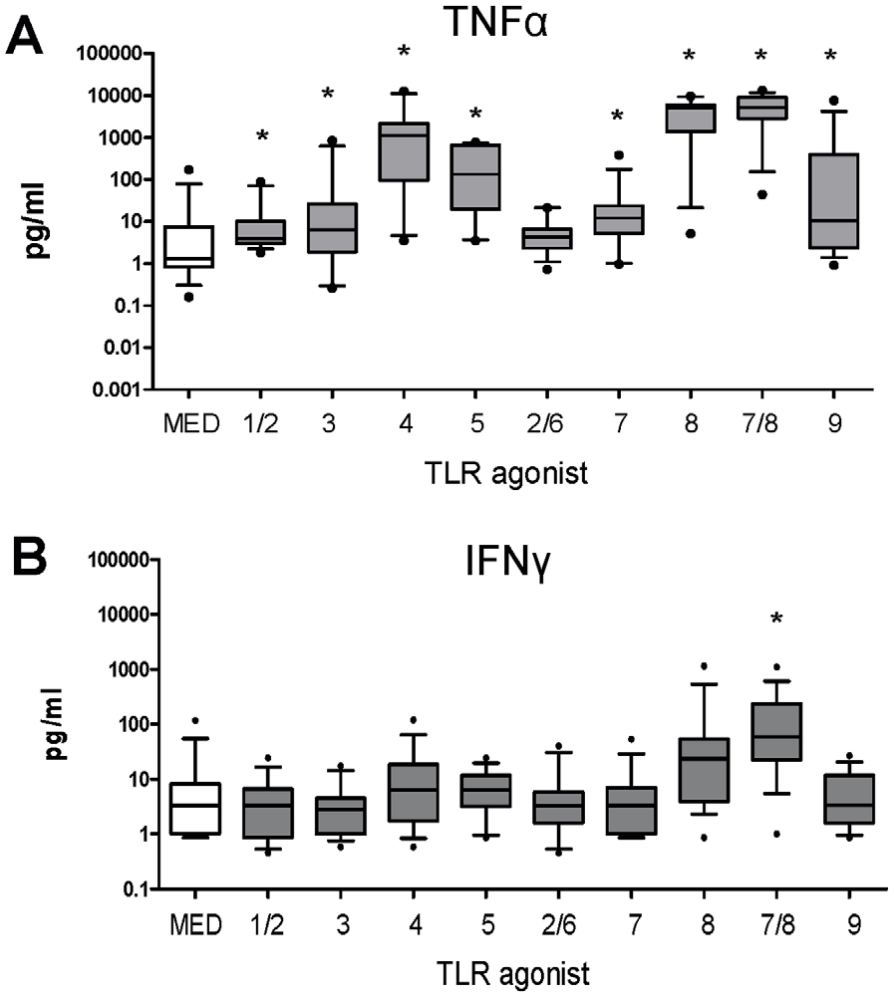
TLR-mediated cytokine production in cord blood. 100 µl of whole cord blood was cultured overnight with each of the TLR agonists and supernatants recovered for measurement of (A) TNFα and (B) IFNγ as described in Materials and Methods (pg/mL) and presented as box and whisker plots illustrating 10 and 90 percentile error bars, n = 12**–**15. Comparisons between cytokine levels in the stimulated samples and the unstimulated samples were analysed using a paired Sign test at 5% significance (* represent significant differences).

**Figure 2:**
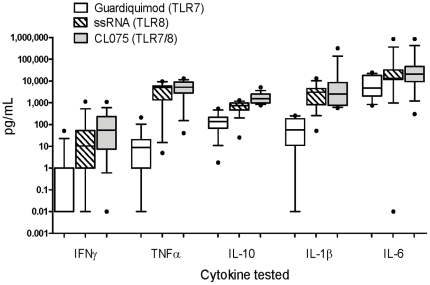
TLR7 and 8 agonist-induced cytokine induction in cord blood. 100 µl of whole cord blood was cultured overnight with each of the TLR agonists and supernatants recovered for measurement of IFNγ, TNFα, IL-10, IL-1β and IL-6 as described in Materials and Methods (pg/mL), n = 12**–**15. Unstimulated values are subtracted from stimulated values and presented as box and whisker plots, n = 12**–**15.

Of note, the efficacy with which TLR agonists induced these cytokines, differed both between agonists and with age. Agonists of TLR4, -5, and, in particular TLR8 (including the combined TLR7/8 agonist) induced the highest levels of most cytokines, with agonists of TLR1/2, -3, -2/6, -7 and -9 eliciting a reduced cytokine response (Table 1 and Figure 1A). The combined TLR7/8 agonist (the thiazoloquinolone CL075) induced the highest levels of TNFα compared to agonists of TLR4 (p = 0.0202) and TLR5 (<0.0001). Moreover CL075 (TLR 7/8) was the only TLR agonist that induced IFNγ in cord blood compared to the unstimulated control (p = 0.002, Table 1 and Figure 1B). In agreement with previous studies [43], the TLR8 agonists (ssRNA and CLO75) induced significantly more cytokine production in cord blood cultures compared to the TLR7 agonist (Gardiquimod™) (Figure 2).

### TLR-mediated IL-6 and IL-1β production is high from birth, while TNFα and IFNγ production peak at 1 month

**Figure 3:**
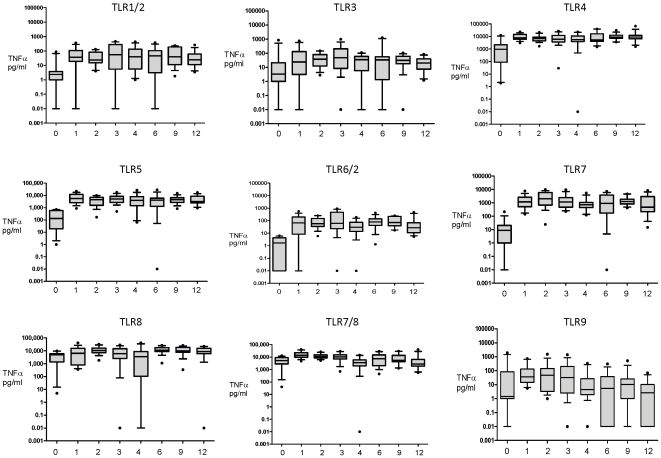
Net TLR-mediated TNFα responses differ between agonists and across the first year of life. 100 µl of whole blood was cultured overnight with each of the TLR agonists at birth, 1, 2, 3, 4, 6, 9 and 12 months of age (x axis) and TNFα cytokine production (y axis) was measured as described in the Materials and Methods (pg/mL). Unstimulated values are subtracted from stimulated values and presented as box and whisker plots, n = 12-15.

**Figure 4:**
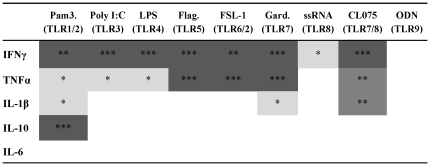
TLR agonists induce greater cytokine production in Gambian newborns at 1 month compared to at birth. 100 µl whole blood was cultured overnight with each of the TLR agonists and cytokine production (pg/mL) in supernatants was measured as described in the Materials and Methods. Comparisons between cytokine levels at birth and 1 month of age were calculated using a non-parametric Mann Whitney test. *(light grey square) p = 0.05 – 0.019, **(medium grey square) p = 0.01 – 0.001, ***(dark grey square) p<0.001. Pam3. = Pam_3_CysSerLys_4,_ Gard. = Gardiquimod.

Each cytokine studied demonstrated a distinct age-dependent profile in response to each TLR agonist during the first year of life. An assessment of effect with age for each cytokine response to each agonist revealed that TNFα responses to FSL-1 (TLR2/6; p<0.001), LPS (TLR4; p = 0.009), Flagellin (TLR5; p = 0.001), Gardiquimod™ (TLR7; p <0.001), ssRNA (TLR8; p<0.001), and CL075 (TLR7/8; p <0.001) all demonstrated age-dependent effects (Table S2A). Graphic representation of these data suggested this effect was due to a predominant increase in TNFα production between cord blood and 1 month of age in response to most agonists (Figure 3). This impression was confirmed by repeating the analysis between ages 1 to 12 months (excluding the cord blood) (Table S2B) and comparing the individual responses between birth and 1 month of age (Figure 4). After the initial increase in TNFα responses by 1 month, TNFα responses to most agonists were stable from 1 month to 12 months of age (Table S2B and Figure 3).

Responses to the TLR8 agonist (ssRNA) and the combined TLR7/8 agonist (CLO75) were notable exceptions to this pattern. The initial increase in reactivity to CLO75 from birth to 1 month of age (p = 0.005), was then followed by a decline from 1 to 12 months of age (trend analysis p<0.001, from a median at 1 month of 13,852 pg/mL, to a median at 12 months of 2,771 pg/mL), though nevertheless remained higher than those for TLR2, -3, -2/6 and -9 (Table S2 and Figure 3). In contrast, TNFα responses to ssRNA (TLR8) did not reveal a significant trend from 1 to 12 months of age (Figure 3). The relatively low TNFα response to Pam3CSK4 (TLR1/2), Poly (I:C) (TLR3) and ODN M362 (TLR9) at birth failed to increase in any age group to 12 months, in fact the TLR9-mediated TNFα response significantly decreased from the 1 month to 12 month age groups (trend analysis p = 0.008, Table S2 and Figure 3).

**Figure 5:**
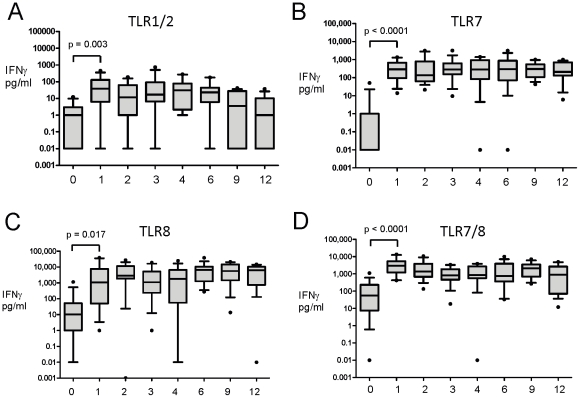
Net TLR-mediated IFNγ responses differ between agonists and across the first year of life. 100 µl of whole blood was cultured overnight with (A) Pam3CSK4 (TLR1/2 agonist), (B) Gardiquimod™ (TLR7 agonist), (C) ssRNA (TLR8 agonist) and (D) CL075 (TLR7/8 agonist) at birth (cord), 1, 2, 3, 4, 6, 9 and 12 months of age (x axis) and IFNγ cytokine production (y axis) was measured in supernatants (pg/mL) as described in Materials and Methods. Unstimulated values are subtracted from stimulated values and data were presented as box and whisker, n = 12**–**15.

While TLR-mediated IFNγ production at birth was very limited (Figure 1 and Table 1), most TLR agonists elicited greater IFNγ production by 1 month of age: Pam3CSK4 (TLR1/2; p = 0.003 (Figure 5A), Poly I:C (TLR3; p<0.001), LPS (TLR4; p<0.001), Flagellin (TLR5; p<0.001), FSL-1 (TLR6/2; p = 0.002), Gardiquimod™ (TLR7; p<0.001; Figure 5B), ssRNA (TLR8; p = 0.017; Figure 5C), CL075 (TLR7/8; p<0.001; Figure 5D), ODN M362 (TLR9; p = 0.066; Figure 4 and Table S2). TLR-mediated IFNγ production then remained stable from 1 to 12 months of age, with the exception of responses to the TLR1/2 agonist that decreased with age (trend analysis; p = 0.003, Figure 5A).

All the TLR agonists studied induced high levels of IL-6 at birth (Table 1) and generally TLR-mediated IL-6 production remained high for the first year of life (Table S2). Similarly, IL-1β and IL-10 production was high at birth in response to TLR4, -5, and -8 agonists and remained high for the first year of life with the exception of the combined TLR7/8 agonist (CL075) that induced less IL-1β with age (Table S2). In accordance with prior reports of an early life bias towards a high ratio of IL-6/TNFα production [40], [41], in our cohort, ratios of TLR-mediated IL-6/TLR-mediated TNFα were initially high at birth (cord) and then decreased with age, reflecting a relatively constant and robust IL-6 production and an increase in TLR-mediated TNFα by 1 month of age (data not shown).

### TLR8 agonists elicit distinct early age-dependent Th1 responses

**Figure 6:**
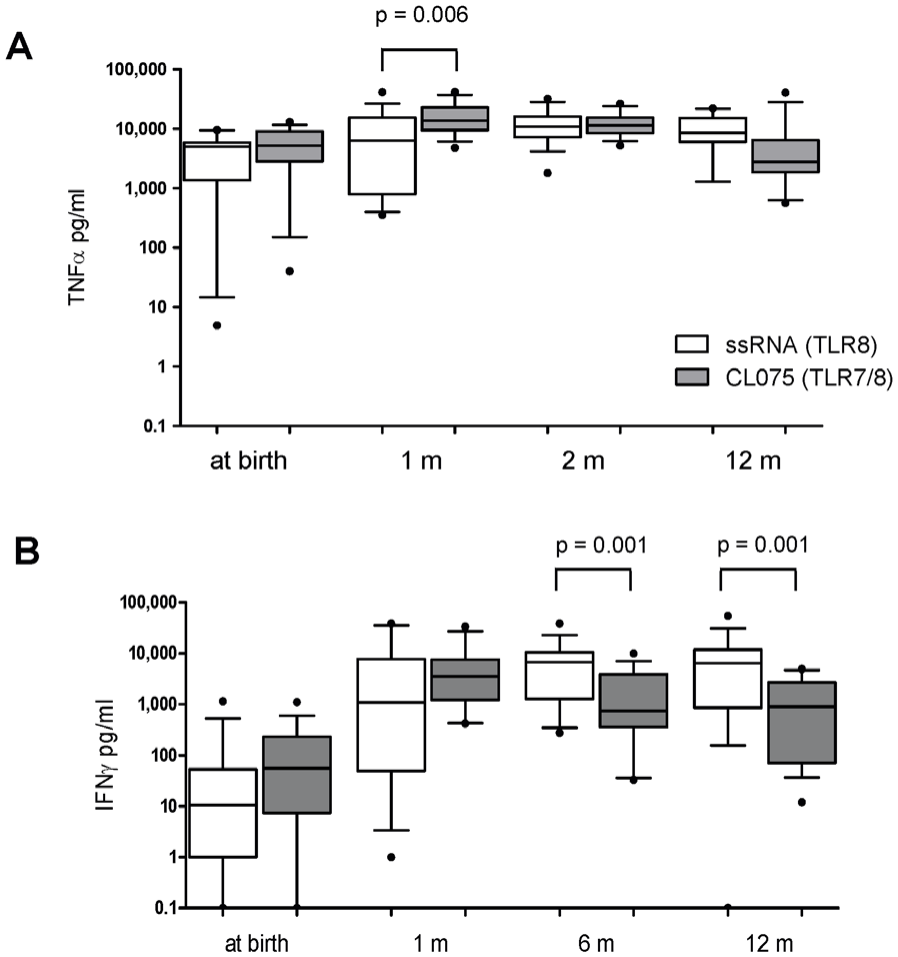
TLR8 agonists induce distinct patterns of TNFα and IFNγ production across the first year of life. 100 µl of whole blood was cultured overnight with ssRNA (TLR8 agonist) and CL075 (TLR7/8) at birth, 1, 2 and 12 months of age. (A) TNFα and (B) IFNγ cytokine (pg/mL) was measured in supernatants as described in the Materials and Methods. Unstimulated values are subtracted from stimulated values and data are presented as box and whisker plots, n = 12**–**15.

In accordance with a prior cord blood study [43], TLR8 agonists induced greater Th1-polarising cytokine responses in early life compared to agonists of other TLRs. CLO75 (TLR7/8) and ssRNA (TLR8) differed with respect to the dynamics across age of cytokine responses. By one month of age, CL075 induced higher concentrations of TNFα than ssRNA (p = 0.006), but by 2 months of age the two agonists induced similar concentrations (p = 0.735) which remained constant up to 12 months (Figure 6A). IFNγ responses to these two agonists were similar from birth up to 6 months of age, from which point ssRNA induced greater levels of IFNγ than CL075 through 12 months of age (p = 0.001) (Figure 6B). In contrast, CL075-induced IL-10 production exceeded that induced by ssRNA at all ages (p<0.05, data not shown).

### TLR8-mediated IFNγ protein production correlates with gene transcription

As the ability to induce IFNγ is of particular importance to host defence and generation of adaptive immunity, we further characterised TLR-mediated IFNγ induction in blood samples from 5 individuals for which longitudinal samples were available from birth, 1 and 12 months of age. In addition, we also measured *IL-12A* and *IL-12B* that encode components of IL-12p70, a cytokine that is important to IFNγ production [48], [49] and to Th1 adaptive immune responses but can be challenging to detect at the protein level. Total RNA was purified from blood samples that were stimulated for 4 hours with Gardiquimod™ (TLR7), ssRNA (TLR8) and CL075 (TLR7/8) and mRNA levels measured by real time PCR. Cytokine mRNAs were significantly up-regulated at each time point for each TLR agonist-stimulated condition compared to the unstimulated control (data not shown).

**Figure 7:**
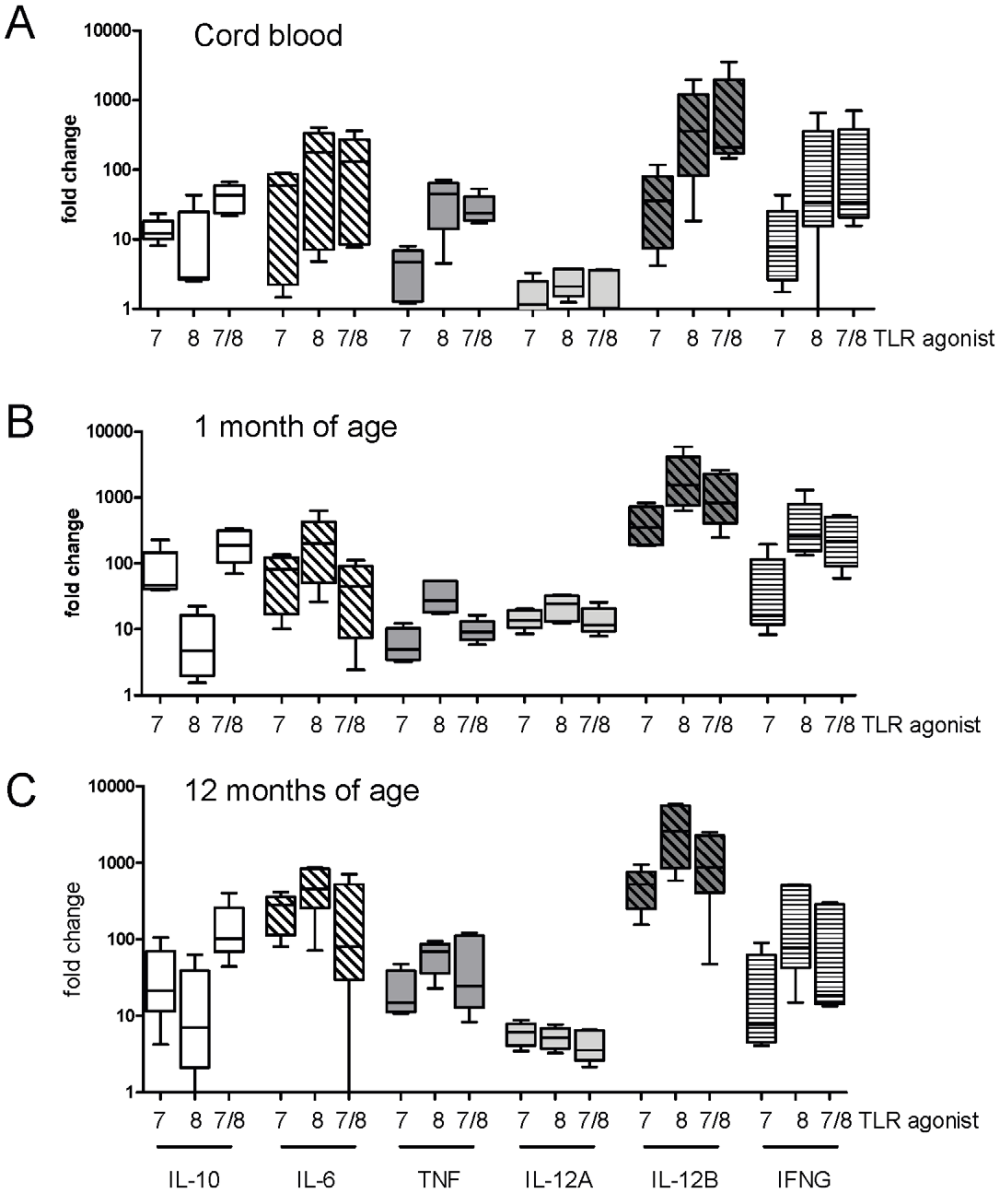
TLR7- and TLR8-mediated cytokine mRNA expression varies between agonists and across the first year of life. 300 µl of whole blood was cultured for 4 hours with Gardiquimod™ (TLR7), ssRNA (TLR8 agonist) and CL075 (TLR7/8) at (A) birth, (B) 1 month and (C) 12 months of age. RNA was purified and cytokine mRNA was quantified by real time PCR as described in Materials and Methods. Ct values were normalised against the housekeeping genes and compared to the unstimulated control (ΔCt) and fold differences of ΔCt values between unstimulated and stimulated cultures were calculated (ΔΔCt). Fold changes are presented as box and whisker plots, n = 5.

In accordance with the protein data, cytokine mRNA expression patterns appeared similar with lower responses to Gardiquimod™ (TLR7) compared to ssRNA (TLR8) and CL075 (TLR7/8) for most cytokines at each age (Figure 7) except *IL-12A* expression that appeared to be expressed at similar levels in response to each of the agonists at a given age (Figure 7). In addition, the pattern of protein concentrations of IL-10 demonstrated similar expression patterns to *IL-10* mRNA with greater responses to CL075 than to ssRNA for all ages (Figure 7).

**Figure 8:**
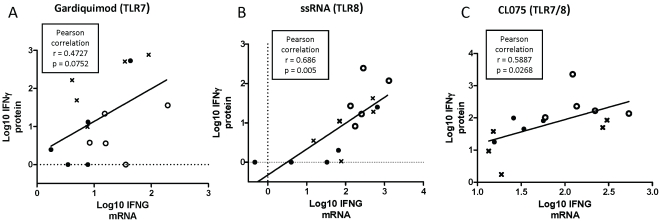
Correlations between ssRNA-induced cytokine mRNA and protein expression. 300 µl of whole blood was cultured for 4 hours with (A) Gardiquimod™ (TLR7), (B) ssRNA (TLR8) and (C) CL075 (TLR7/8). RNA was purified and cytokine mRNA was quantified by real time PCR as described in Materials and Methods. Comparisons between IFNγ protein and *IFNG* mRNA levels were calculated where stimulated values (Ct and cytokine concentrations) were divided by unstimulated values. The values were log transformed and compared using Pearson correlation test, linear regression line presented in graph, n =  5. At birth (black circles), 1 month (open circles), or 12 months (crosses) of age.

Correlations between mRNA expression levels and protein levels for the same cytokines were analysed as pooled ages. In accord with greater IFNγ response to TLR8 versus TLR7 agonists, significant correlations were noted between TLR agonist-induced mRNA expression of *IFNG* and IFNγ protein levels induced by ssRNA (p = 0.005; Figure 8B) and CL075 (p = 0.027; Figure 8C), but not Gardiquimod™ (p = 0.075; Figure 8A). Of note, TLR-mediated increases in *IL12A* and *IL12B* mRNA also correlated with TLR-mediated IFNγ protein production, including ssRNA-induced *IL-12A* and *IL-12B* expression (p = 0.017, p = 0.001 respectively) as well as CL075- and Gardiquimod™-induced *IL-12B* expression (p = 0.027 and p = 0.035, respectively). For the other cytokines studied, the ssRNA- and CL075-induced cytokine mRNA levels at 4 hours of stimulation did not correlate with the corresponding cytokine protein levels at 24 hours.

**Figure 9:**
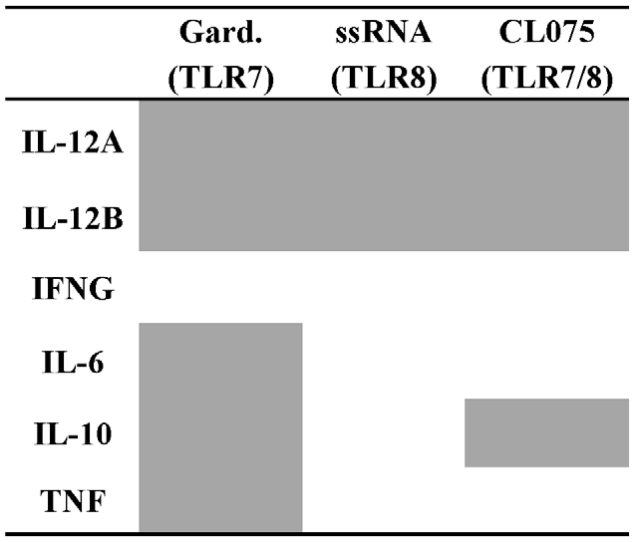
Age-dependent effects of cytokine transcription in response to TLR agonists. 300 µl whole blood was cultured for 4 hours with Gardiquimod™ (TLR7 agonist), ssRNA (TLR8 agonist) and CL075 (TLR7/8 agonist) at birth (cord), 1 and 12 months of age and *IL-12A, IL-12B, IFNG,IL-6, IL-10* and *TNF* cytokine mRNA gene transcription was measured. Ct values were normalised against the housekeeping genes and compared to the unstimulated control (ΔCt). Comparisons of responses from birth, 1 and 12 months of age were made using the Kruskall Wallis test. Grey squares represent significant effects with age, p>0.05. Gard. = Gardiquimod.

Levels of TLR-mediated *IL-12A* and *IL-12B* mRNA expression were age-dependent for all three TLR agonists whereas *IFNG* showed similar levels between birth, 1 and 12 months of age (Figure 9 and Figure 7). In response to CL075 (TLR7/8 agonist), expression of *IL-12A* increased from birth to 1 month of age (p = 0.008) but subsequently decreased by 12 months of age (p = 0.008). These mRNA patterns corresponded to a similar pattern of IFNγ protein production by the same agonist (comparing for the same subjects): birth vs. 1 month of age (p = 0.002), and 1 month vs. 12 months of age (p = 0.018). Gardiquimod™ also demonstrated age-dependent expression of mRNAs encoding *IL-10, IL-6* and *TNF* mainly due to low expression levels at birth that increased with age: birth to 1 month of age: IL-10 (p = 0.008), and birth to 12 months of age: IL-10 p = 0.3095, IL-6 p = 0.032, TNF p = 0.008 (Figure 9 and Figure 7).

## Discussion

Our study evaluated the ontogeny of TLR-mediated *in vitro* whole blood cytokine responses during the first year of life in infants from The Gambia employing agonists of TLRs 1-9 and blood samples from 8 different age groups. We found that cytokine responses of Gambian infants varied between TLR agonists, with the greatest responses to TLR8 agonists (ssRNA and CL075), substantial responses to TLR4 and TLR5 agonists, and relatively weak responses to TLR1, 2, 3, 7 and 9 agonists at all ages. Thus the ontogeny of responses is TLR-specific with distinct differences across age groups in the first 12 months of life. Most of the previous studies exploring TLR-mediated responses of newborns have focused solely on comparisons between cord blood and adults [4], [34], [40]. The few studies that have also examined infant responses are limited to resource-rich setting and did not include the wide range of agonists tested in our study [37], [39], [44], [45].

We found that CL075 (TLR7/8) was the only TLR agonist to induce IFNγ at birth but that in general, cord blood pro-inflammatory/Th1-polarizing cytokine responses (i.e., TNFα and IFNγ) were lower than at later age groups, in accordance with prior studies [4], [34], [39]. In contrast, TLR-mediated neonatal IL-6, IL-1β and IL-10 production was higher or similar at birth compared to later age groups, suggesting that neonatal TLR-mediated responses are biased towards acute phase (IL-1β and IL-6) and anti-inflammatory (IL-10) cytokines. We have previously shown in 5-day cultures that IL-10 and IL-6 responses to mycobacterial antigens were present at birth [50]. It has been speculated that the bias towards high IL-10 production may reflect the need to dampen potentially over-exuberant responses to the numerous new antigens to which an infant is exposed in early life [51]. Greater IL-6 to TNFα ratios have been found in cord blood compared to adults, both in response to *in vitro* stimulation [41] and with respect to basal serum levels during the first week of life [40]. Our findings indicate a similar polarisation in our cohort with greater IL-6 to TNFα ratios at birth compared to 12 months of age. Relatively high IL-6 production in newborns likely contributes to initiation of an acute phase response at birth that may serve to clear perinatally-acquired microbes [52]. In addition, IL-6 enhances both differentiation of Th17 cells and production of IL-17, and thereby may enhance neutrophil- and antimicrobial peptide-based host defence at neonatal mucosal and epithelial barriers [52**]**–[****56]. That this neonatal bias towards TLR-mediated IL-6 and IL-10 responses is evident *in vivo* was recently demonstrated in a field trial of alum-adjuvanted pneumococcal conjugate vaccine [3].

For most TLR agonists the major effect of age occurred within the first month of life during which time TLR-mediated TNFα and IFNγ cytokine production increased, suggesting early maturation in the ability to mount these responses. Whether this maturation in cytokine production reflects changes in the adenosine system that serves to limit production of TNFα in human newborn cord blood [41], will be the subject of future studies. The only other study that reported responses in 1 month old infants was conducted by Belderbos *et al* in Holland, which also showed increased pro-inflammatory responses (e.g., IL-12p70 and IFNα) from birth to 1 month of age in response to TLR3, -4, -7 and -9 agonists but similar levels of IL-10 production in response to TLR3, -4 and -9 [39]. That study found that loxoribine (TLR7)-induced IL-10 responses increased by 1 month of age, in accord with our observation of a borderline increase at 1 month of age (p = 0.05) using the TLR7 agonist Guardiquimod™. A Belgian study by Ngyuen *et al* also found that LPS-induced cord blood production of IL-10 and IL-6 was greater than in later age groups (6-9 months and 12 months of age). Taken together, our current study and those by Belderbos *et al* [39], and Vosters *et al* [44] indicate that for most TLR agonists, IL-10 production is largely similar from birth through to 18 months of age.

A comparison of the few studies of infant TLR function [37], [39], [44], [45] and our current study indicates that Gambian infants have broadly similar TLR-mediated responses to those found in a Western European environment. However, Ngyuen *et al* studying a Belgian cohort found a slower postnatal increase in TNFα production in response to LPS from birth compared to our study, such that adult levels were not reached until 6 months of age [37], whereas our Gambian cohort demonstrated LPS-induced TNFα responses that peaked at 1 month of and then remained stable to 12 months of age. Earlier maturation of LPS-induced TNFα responses in Gambian infants than in the European infants [37], [39], [45] may reflect more rapid polarisation to Th1 responses in a resource-poor setting, in accord with the hygiene hypothesis [13], [57], [58], and may also suggest that TLR4 agonists, currently used in several vaccine formulations (e.g., MPL[59]), may be useful vaccine adjuvants in early infancy.

Although TLR8 agonist-induced cord blood TNFα responses have been shown to be greater than TNFα responses to agonists of TLRs 1**–**7 [43], little is known regarding cytokine responses to TLR8 agonists such as ssRNA (TLR8 only) and CL075 (TLR7/8 agonist) during the first year of life. Our study confirmed the greater responsiveness to TLR8 agonists in cord blood and extended the finding by demonstrating that TLR8 agonists were the predominant inducers of pro-inflammatory cytokines up to 12 months of age in Gambian infants. We also found that the combined TLR7/8 agonist (CL075) induced more TNFα than the TLR7 selective agonist Gardiquimod™ or the TLR8 selective agonist ssRNA up to 1 month of age. By 12 months of age however, there was a trend towards reduced TNFα production in response to the combined agonist compared to the single TLR8 agonist. Likewise, IFNγ production was similar between the two agonists up to 6 months of age after which CL075-induced IFNγ diminished relative to ssRNA-induced IFNγ and remained lower up to 12 months of age. It should be noted however, that in addition to differences between TLR7 and TLR8 selectivity, differences in biochemical structures between ssRNA and the low-molecular weight thiazoloquinolone, CL075 may affect TLR-independent variables (e.g., solubility, protein binding and cell penetration) that may also contribute to distinct bioactivities. The correlations between *IFNG* mRNA and IFNγ protein in response to ssRNA and CL075 indicate that the ontogeny of TLR-mediated IFNγ production is manifest at the transcriptional level. Given the importance of IL-12 to IFNγ production [49], [60], [61] and the observed correlations of TLR-mediated IFNγ protein with *IL12B* message, TLR8-mediated IFNγ responses may also involve activation of *IL-12B* (that encodes for IL-12p40)[49]. However, expression of *IL-12A* (that encodes for IL-12p35) was very low at all three ages tested, in agreement with Western European studies that demonstrate deficiency in TLR-mediated IL-12p35 production by neonatal DCs, contributing to impaired neonatal IFNγ responses [62]. Analysis of TLR-mediated production of additional cytokines that may influence adaptive immune responses, including IL-4 (Th2) and IL-17 (Th17), is of importance and should be included in future studies.

Overall, characterizing the ontogeny of innate immune responses may eventually inform selection of adjuvant vaccine formulations that are tailored for certain age groups. Indeed, recent studies highlight that appropriate immunologic signatures can predict vaccine efficacy [17], [63]. To the extent that TNFα and IFNγ may be markers of strong Th1-polarising responses required for induction of cell-mediated immunity, and that the whole blood *in vitro* responses measured in our study may reflect those that would pertain *in vivo*, our data suggest that vaccine adjuvants based on TLR4, -5 or -8 agonists, may be particularly effective in protecting newborns and infants against pathogens requiring cell-mediated immunity.

## Supporting Information

Table S1 Summary of the TLR agonists used in the study.
(DOC)

Table S2 **Analysis for age-dependent evolution of cytokine responses to TLR agonists.** 100 µl of whole blood was cultured overnight with Pam3CSK4 (TLR1/2), Poly (I:C) (TLR3), LPS (TLR4), Flagellin (TLR), FSL-1 (TLR6/2), Gardiquimod™ (TLR7), ssRNA (TLR8) and CL075 (TLR7/8) or ODN M362 (TLR9) at birth (cord), 1, 2, 3, 4, 6, 9 and 12 months of age. IFNγ, TNFα,IL-1β,IL-10and IL-6 cytokine concentrations (pg/mL) were measured in supernatants as described in Materials and Methods. Comparisons of responses were made (A) from birth to 12 months of age and (B) from 1 to 12 months of age by comparing the proportions of values above and below the median using Fisher's exact test. All grey squares represent significant effects with age (p<0.05) while dark grey squares indicate the effects with age corresponding to a significant decline in cytokine production from 1 to 12 months of age using trend analysis.
(DOC)
