# Supplementary material for: Age-Dependent Maturation of Toll-Like Receptor-Mediated Cytokine Responses in Gambian Infants
Source: PLoS One. 2011 Apr 13;6(4):e18185. doi: 10.1371/journal.pone.0018185 (PMC3076452; doi:10.1371/journal.pone.0018185)
Supplement: Table S1 — Summary of the TLR agonists used in the study. (DOC) [file pone.0018185.s001.doc]

**Table S1: Summary of the TLR agonists used in t**he study

| **TLR agonist name** | **TLR receptor** | **Supplier** | **Catalogue number** | **Final Concentration** |
| --- | --- | --- | --- | --- |
| Pam_3_CysSerLys_4_ | 1/ 2 | InvivoGen | tlrl-pms | 1µg/mL |
| Poly I:C | 3 | InvivoGen | tlrl-pic | 100µg/mL |
| LPS | 4 | InvivoGen | tlrl-eklps | 1µg/mL |
| Flagellin | 5 | InvivoGen | tlrl-stfla | 10µg/mL |
| FSL-1 | 6/ 2 | InvivoGen | tlrl-fsl | 10µg/mL |
| Guardiquimod | 7 | InvivoGen | tlrl-gdq | 10µg/mL |
| ssRNA | 8 | InvivoGen | tlrl-lrna40 | 10µg/mL |
| CL075 | 7/ 8 | InvivoGen | tlrl-c75 | 10µg/mL |
| ODN M362 (Type C stimulates both NFκB and IFNα pathways) | 9 | InvivoGen | tlrl-hodnc | 1µM |
