# Supplementary material for: Age-Dependent Maturation of Toll-Like Receptor-Mediated Cytokine Responses in Gambian Infants
Source: PLoS One. 2011 Apr 13;6(4):e18185. doi: 10.1371/journal.pone.0018185 (PMC3076452; doi:10.1371/journal.pone.0018185)
Supplement: Table S2 — Analysis for age-dependent evolution of cytokine responses to TLR agonists. 100 µl of whole blood was cultured overnight with Pam3CSK4 (TLR1/2), Poly (I:C) (TLR3), LPS (TLR4), Flagellin (TLR), FSL-1 (TLR6/2), Gardiquimod™ (TLR7), ssRNA (TLR8) and CL075 (TLR7/8) or ODN M362 (TLR9) at birth (cord), 1, 2, 3, 4, 6, 9 and 12 months of age. IFNγ, TNFα,IL-1β,IL-10and IL-6 cytokine concentrations (pg/mL) were measured in supernatants as described in Materials and Methods. Comparisons of responses were made (A) from birth to 12 months of age and (B) from 1 to 12 months of age by comparing the proportions of values above and below the median using Fisher's exact test. All grey squares represent significant effects with age (p<0.05) while dark grey squares indicate the effects with age corresponding to a significant decline in cytokine production from 1 to 12 months of age using trend analysis. (DOC) [file pone.0018185.s002.doc]

**Table S2: Summary of age-dependent effects of cytokine responses to TLR agonists**

**A: Effect with age from birth to 12 months of age**

|  | **Pam3CSK4** | **Poly I:C** | **LPS** | **Flag** | **FSL-1** | **Gard** | **ssRNA** | **CL075** | **ODN** |
| --- | --- | --- | --- | --- | --- | --- | --- | --- | --- |
|  | **TLR1/2** | **TLR3** | **TLR4** | **TLR5** | **TLR6/2** | **TLR7** | **TLR8** | **TLR7/8** | **TLR9** |
| **IFNγ** |  |  |  |  |  |  |  |  |  |
| **TNFα** |  |  |  |  |  |  |  |  |  |
| **IL-1β** |  |  |  |  |  |  |  |  |  |
| **IL-10** |  |  |  |  |  |  |  |  |  |
| **IL-6** |  |  |  |  |  |  |  |  |  |

**B: Effect with age from 1 to 12 months of age (excluding responses at birth)**

|  | **Pam3CSK4** | **Poly I:C** | **LPS** | **Flag** | **FSL-1** | **Gard** | **ssRNA** | **CL075** | **ODN** |
| --- | --- | --- | --- | --- | --- | --- | --- | --- | --- |
|  | **TLR1/2** | **TLR3** | **TLR4** | **TLR5** | **TLR6/2** | **TLR7** | **TLR8** | **TLR7/8** | **TLR9** |
| **IFNγ** |  |  |  |  |  |  |  |  |  |
| **TNFα** |  |  |  |  |  |  |  |  |  |
| **IL-1β** |  |  |  |  |  |  |  |  |  |
| **IL-10** |  |  |  |  |  |  |  |  |  |
| **IL-6** |  |  |  |  |  |  |  |  |  |
